# Dimensions of uncertainty communication: What is conveyed by verbal terms and numeric ranges

**DOI:** 10.1007/s12144-022-03985-0

**Published:** 2022-11-12

**Authors:** Karl Halvor Teigen

**Affiliations:** Department of Psychology, University of Oslo, Oslo, Norway

**Keywords:** Verbal probabilities, Prediction intervals, Directionality, Credibility

## Abstract

The paper reviews two strands of research on communication of uncertainty that usually have been investigated separately: (1) Probabilities attached to specific outcomes, and (2) Range judgments. Probabilities are sometimes expressed by verbal phrases (“rain is likely”) and at other times in a numeric format (“70% chance of rain”), whereas range judgments describe the potential amounts expected (“1–4 mm of rain”). Examination of previous research shows that both descriptions convey, in addition to the strength of expectations, pragmatic information about the communicative situation. For instance, so-called verbal probability expressions (VPE), as *likely*, *unlikely, a chance,* or *not certain* give some, albeit vague, probabilistic information, but carry in addition an implicit message about the sources of uncertainty, the outcome’s valence and severity, along with information about the speakers’ attitudes and their communicative intentions. VPEs are *directional* by drawing attention either to an outcome’s occurrence (“it is possible”) or to its non-occurrence (“it is doubtful”). In this sense they may be more informative than numbers. Uncertainties about outcomes in a distribution (continuous quantities) are alternatively expressed as interval estimates. The width of such intervals can function as a cue to credibility and expertise. Incomplete, one-sided intervals, where only one boundary is stated, imply directionality. “More than 100 people” suggests a crowd, while “less than 200” implies a shortfall. As with VPEs, directionally positive intervals are more frequent, and perhaps more neutral than negative ones. To convey expectancies and uncertainty in a balanced way, communicators may have to alternate between complementary frames.

## Introduction

Past facts and future outcomes are rarely known exactly, and have to be expressed in tentative or approximate ways (for an overview, see Ferson et al., 2015). We say that the pandemic has claimed “more than” 6 million lives worldwide, and that the global temperature “could” increase by 4 degrees within this century. Such statements contain explicit factual information that may be true or false, although it is not always easy to verify or falsify a vague prediction. But they also convey a more implicit, pragmatic message, for instance about the speakers’ expertise, their concerns and communicative intentions.

In a context of quantifiable events (magnitudes and amounts), we can distinguish between two approaches: (1) Statements about one specific outcome, for instance a “best guess”, modified by a probabilistic quantifier, which can be numeric or verbal (“2 mm of rain is *likely”*). We may call this *the probabilistic approach*. (2) Alternatively, a statement can express a set of potential outcomes (“we will have *0–4 mm* of rain”). We may call this the *range approach.* The two descriptions are complementary by indicating degree of probability, in the first case, and an uncertainty interval, in the second. They can be combined into a confidence interval (“*0–4 mm* of rain is *very likely*”).

Which messages do such statements convey to the public? We present in this article a structured overview of research that has examined the interpretations of such statements by the receivers. This research has typically studied aspects of one approach separate from the other, whereas the present survey will present them side by side, to gain insights in how similar they are and how they differ.

We are not in this article concerned with how such estimates are arrived at, or how accurate they are, but with the meanings they convey to listeners or readers, dependent on communication format. Some meanings are explicit and factual. An outcome that has “a 70% chance” of occurring signifies a stronger expectation than an outcome that is merely “possible”. Another speaker may prefer to say it is “not certain”. The two last options may indicate similar expectations, but differ as to how they are described, or *framed*. Frames differ by directing listeners attention towards complementary aspect of a situation. They may, to use the metaphor of Sher and McKenzie (2006) “leak” information of a pragmatic nature. A”half empty” glass is compared to a full glass, whereas a “half full” glass is compared to an empty one (McKenzie & Nelson, 2003). Words, and perhaps also numbers, are rarely completely neutral but might contain clues to speakers’ beliefs, concerns, and preferences. They may further indicate the origin of knowledge and its limitations, the speakers’ credibility, and influence evaluations and decisions. Such effects are at times intended but might create a problem for professionals who are presumed to express their estimates in an objective and neutral fashion.

The research surveyed here is not restricted to a specific domain like medical risks or weather forecasts, but applies to most settings where outcome uncertainty prevails. Most research on uncertainty communication has addressed the issue of accuracy, and how well recipients’ understandings are aligned with what an analyst or communicator have in mind (for recent overviews, see Dhami & Mandel, 2022; European Safety Authority et al., 2018; Van Der Bles et al., 2019). The present paper highlights, in contrast, pragmatic meanings of the phrases and formats used, that arise from their conversational implications, whether intended or not intended by the communicator. Both approaches can convey several overlapping facets of such “surplus” meanings, as sketched in Table 1 and briefly discussed in the subsections of the present article. The present author’s interest in these connotations dates back to a more speculative paper published 35 years ago (Teigen, 1988), as a reaction to a rather narrow view of verbal probabilities in the field of judgment and decision making, where words were viewed as an imperfect substitute for probabilistic numbers. By now the literature has grown wide and varied and might deserve an updated review of relevant research conducted by the original author and his associates as well as by a large number of other scholars.

**Table 1:** Facets of meaning conveyed by verbal and numeric statements about uncertain outcomes

Verbal probabilities of specific outcomes			Uncertain outcomes as numeric ranges		
Section		Examples	Section		Examples
1	Degrees of probability	Numeric translations of verbal phrases “Likely” means 70%	1	Degrees of uncertainty	Interval width 40–60 cm vs. 20–80 cm increase in sea level
2	Hedging	Politeness and understatements “It is possible”	2	Hedging	Approximate estimates “About”, use of round numbers
3	Sources of probability	External vs. internal “it is uncertain” vs. “I am uncertain”	3	Sources of uncertainty	External variability vs. imprecise knowledge
4	Expertise and credibility	Numbers suggest expertise The confidence heuristic	4	Expertise and credibility	Preciseness suggests expertise (when plausible)
5	Valence	Positive vs. negative outcomes “A hope” vs. “a risk”	5	Comparative judgments	Exclusive vs. inclusive ranges “More than–less than” vs. “at least–at most”
6	Directionality of VPE	Framing with verbal expressions “Likely” vs. “not completely certain”	6	Directionality of incomplete ranges (upper or lower bounds)	Framing with single bounds “At least X” vs. “at most Y”
			7	Which of multiple or continuous outcomes correspond to a VPE	The “Which Outcome” approach Extremity and centrality effects

## Probabilities of specific target outcomes

In this section we are concerned with probability expressions of target outcomes that can be conceived as categorical: a patient is infected by the flu (or not infected), we will have rain tomorrow (or no rain). Such outcomes can be assigned numeric probabilities between 0 and 100% or be qualified by so-called by verbal probability expressions (VPE) such as *unlikely*, *possibly,* and *almost certain*. Such estimates are also used to describe the status of other knowledge items (like theories and hypotheses), which may be described as *doubtful*, *likely*, or *uncertain*, but more rarely given numeric estimates.

Probabilities of specific target outcomes are ideally expressed in a numeric format. Numbers are precise and unambiguous; they can be derived from statistics and formal computations and can, in turn, be used as input to further calculations. But numeric estimates may also be attached to outcomes that are not calculable in a formal sense but are derived from human judgments. Studies of such judgments have shown that people’s intuitive (estimative) numeric probabilities are poorly calibrated and show “overconfidence” when compared to actual hit rates (Moore & Healy, 2008; Moore et al., 2016). They may also be inconsistent, due to people’s neglect of formal rules for distributional probabilities, like additivity and the conjunction rule, suggesting that they reflect subjective indicators of support (Tversky & Koehler, 1994) rather than objective frequencies. Even unbiased numeric estimates can be criticised for appearing more scientific and exact than warranted.

Verbal statements have the advantage of being more flexible, less pretentious and more familiar, and hence perhaps more readily expressed and understood. It seems both easier and more adequate for a physician to say that it is *likely* you have the flu than to come up with a numeric estimate of, say, a 70% probability. According to the so-called “communication preference paradox” (Erev & Cohen, 1990), speakers prefer to use verbal statements, whereas listeners prefer to receive numbers. However, when probabilities are imprecisely known, the preference for numbers is reduced (Juanchich & Sirota, 2019).

Words obey their own linguistic logic that makes them more universally applicable than numbers. The vocabulary people use to express their expectations is rich and flexible. It includes verbs (I *believe*, I *suspect*, something *may* or *will* happen), adjectives and adverbs (*likely, possible*, *perhaps*) and nouns (a *hope*, a *risk*, a *chance*). These may in turn be modified by intensifiers (*a good chance*, *very likely*), comparatives *(more likely*), and negations (*impossible, not certain, beyond a reasonable doubt*). A complete list of alternative ways of expressing expectancies in ordinary language has yet to be attempted. For overviews of approaches and findings, see Budescu and Wallsten (1995), Teigen and Brun (2003a), Collins and Hahn (2018), and Juanchich et al. (2019) In the present section, we briefly identify and discuss six prominent facets of meaning that verbal phrases may convey.

### Levels and degrees of probability

Discussions of verbal phrases presume that their main function is to indicate levels and degrees of probability. A *likely* outcome is assumed to have a high probability of occurrence (corresponding to probabilities around 70%), while *unlikely* outcomes are assumed to occur rarely (equivalent to 30% or less). Tables of verbal phrases with corresponding numeric probabilities have been constructed by panels of experts in various domains (security and military intelligence, climate research, medicine and health, marketing and accounting, among others) to simplify and standardise the communication of uncertainty to decision makers and the public at large. Such scales are typically symmetrical and consist of 5–9 standard steps, as illustrated in Table 2.

**Table 2:** Approximate probability scales recommended by EFSA (European Food Safety Authority), IPCC (Intergovernmental Panel on Climate Change), and NATO (North Atlantic Treaty Association). Adapted from European Food Safety Authority et al. (2018, Table 4), and Mastrandrea et al. (2010), and Allied Joint Doctrine for Intelligence Procedures AJP-2.1 (in Irwin & Mandel, 2020)

EFSA (2018)		IPCC (2010)		NATO (2016)	
Probability term	Subjective probability range	Term	Likelihood of outcome	Verbal statement	Numerical assessments
Almost certain	99–100%	Virtually certain	99–100%		
Extremely likely	95–99%				
Very likely	90–95%	Very likely	90–100%	Highly likely	More than 90%
Likely	66–90%	Likely	66–100%	Likely	60–90%
About as likely as not	33–66%	About as likely as not	33–66%	Even chance	40–60%
Unlikely	10–33%	Unlikely	0–33%	Unlikely	10–40%
Very unlikely	5–10%	Very unlikely	0–10%	Highly unlikely	Less than 10%
Extremely unlikely	1–5%				
Almost impossible	0–1%	Exceptionally unlikely	0–1%		

Unfortunately, these scales are rarely empirically validated to ascertain that they are understood as intended. When validations are attempted, they often show large gaps between people’s responses and the prescribed meanings (Berry, 2004; Budescu et al., 2014; Wiles et al., 2020). However, ordered sets of terms may be helpful by suggesting levels. Even an elusive term as *possible* makes sense when wedged between *likely* and *unlikely*. In addition, several authors have recommended use of verbal-numerical (V–N) combinations, which they think will lead to better understanding than either format taken in isolation. We might for instance learn that temperatures in the future are *likely* (> 66% chance) to reach 3 degrees, or that the chances of a terrorist attack are *remote* (< 10%). Such combined expressions give a rough guide to what the phrases mean, as well as offering an informal explanation of the numbers (Barnes, 2016; Harris et al., 2017; Wintle et al., 2019; Zhang et al., 2019). But it is difficult to legislate word use. Even in contexts where such standards are imposed, as in the IPCC reports on climate change (Mastrandrea et al., 2010), other terms not on the list, for instance, *can* and *could*, are used repeatedly to draw attention to expectancies that are not formally defined. One major limitation of such scales, which rarely is discussed, is that they seem to assume that all uncertainties are binary (Teigen et al., 2022a). For instance, *p* = 50% is defined as equivalent to “even chance”. But in a situation with several equal options, as in a raffle where all players have one ticket each, an “even chance” must be much less than 50%.

Empirically based “translations” of verbal phrases (VPE) into corresponding numbers can be obtained by asking people to estimate which probabilities they associate with selected verbal terms, in isolation or in contexts (e.g., Beyth-Marom, 1982; Clarke et al., 1992; Lichtenstein & Newman, 1967; Reagan et al., 1989; Theil, 2002). These studies show general agreement at the group level, but large inter-individual variations, wider than most people seem aware of (Amer et al., 1994; Brun & Teigen, 1988). Some words are worse than others, and can give rise to misunderstandings, as when *not certain* is translated by probabilities ranging from 5 to 70% (Bryant & Norman, 1980). Other VPEs that are too indeterminate to be useful, are “most probably”, “it cannot be ruled out”, and “risk remains” (Barnes, 2016). Only phrases denoting midpoints (“about as likely as not”) or endpoints (“impossible” and “certain”) on the probability dimension are unambiguous.

The consensus seems to be that VPEs are generally *vague*, and characterise a fuzzy range of probabilities, rather than specific points on the 0–100% scale. Attempts have accordingly been made to map the numeric meanings of selected phrases as distributions with a characteristic shape and peak (Budescu & Wallsten, 1985, 1995; Wallsten et al., 1986). Mapping the “membership function” of a single phrase requires a large number of responses from each participant, which in addition to the time and effort involved, demands considerable insight in the usage of such terms in a variety of contexts.

The probabilistic vagueness of verbal terms, and their resistance against standardization, have made researchers sceptical about their role in professional communication (Barnes, 2016; Doupnik & Richter, 2003; Nakao & Axelrod, 1983). But even those who advise against such terms, are likely (sic) to use a variety of such phrases in less guarded moments, for instance when drawing “tentative” conclusions, or characterising hypotheses as “possible” or “very likely”*.* Perhaps scientists’ long-standing fascination with numerical probabilities (Gigerenzer & Murray, 1987), has made them neglect other facets and communicative functions of these phrases.

### Hedging

The vagueness of verbal phrases is not always a liability. The Renaissance essayist Michel de Montaigne felt such terms were praiseworthy: “I love those words or phrases which mollifie and moderate the temerity of our propositions: ‘*It may be: Perhaps: In some sort: Some: It is said: I think*,’ and such like” (Montaigne, 1885, p. 528). By adding hedges, a speaker (or a writer) will appear more modest and admit the possibility of alternative views.

Moderate language has important social functions. Linguistic politeness theory (Brown & Levinson, 1978) assumes that people use caution and understatements as a face-saving device. Tactful remarks, especially about a sensitive issue, protect hearers from being offended and speakers from being blamed for potential mistakes. Considerate speakers chose a weaker VPE to sugar-coat a negative and potentially threatening message; for instance, doctors may say that the tumour is *possibly* malignant when they think that this is *probably* the case, and save *probably* for cases when they are, in fact, quite *certain* (Bonnefon & Villejoubert, 2006). Such understatements are often correctly identified by patients (Juanchich & Sirota, 2013), but can lead to misunderstandings if taken literally (Bonnefon et al., 2011).

We have here the seeds of a paradox. On one side, politeness considerations require speakers to use nonprovocative language. At the same time, strong language may be needed to alert the public to hazards with potentially severe consequences (cf. warning labels on tobacco products). The meanings of a verbal term will accordingly change in different communication settings. leading to discussions about a “severity effect” and the use of verbal phrases under face-threatening conditions (Harris & Corner, 2011; Holtgraves & Perdew, 2016; Patt & Schrag, 2003).

### Sources of probability

People’s expectations have many origins and can be attributed to a variety of sources. They may originate in external processes, like causal mechanisms and statistical trends, or reflect internal states of belief in the mind of a human speaker. Similarly, their lack of certainty may be attributed to external variability and randomness, or simply be due to insufficient knowledge.

The distinction between external and internal conceptions of probability has a long tradition within the philosophy of science (Hacking, 1975). External views include the classical and frequentist accounts, where probabilities can be assigned to outcomes by formal procedures; these are often called *aleatory* due to their origin in games of chance. But people do not always base their probability statements on objective statistics, but use them also for the outcomes of unique events, like the chances of a specific candidate to win a political election. Such estimates of probabilities (numerical or verbal) make sense as reflecting the strength of causal tendencies operating for or against the target outcome. The causal view has been discussed as a *dispositional* or a *propensity* account of probabilities (Gillies, 2016; Popper, 1959), and may come closer to how lay people think of chances in daily life (Kahneman & Tversky, 1982; Keren & Teigen, 2001; Løhre, 2018).

Alternatively, probabilities may be construed to reflect degrees of belief or knowledge (or lack of it). According to this *epistemic* (sometimes called Bayesian) account, high probabilities indicate strong beliefs or high degree of confidence in a proposition, whereas low probabilities express weak beliefs or lack of knowledge.

These external or internal sources of expectancies are reflected in the words used to describe them. So-called epistemic verbs, like to *believe*, *assume*, *suspect*, *guess*, or *doubt* describe the speaker’s states of knowledge. Sentences with such verbs require a person as their grammatical or logical subject. It takes *somebody* to believe, or to doubt, that the pandemic will come to an end in 2022. In contrast, auxiliary verbs, like *will* and *could*, describe more “objective”, externally grounded expectancies. The coronavirus *can* persist regardless of what experts think. Statements of this type take impersonal rather than personal pronouns. *It* (not I) can happen, *I* (not it) believe it will.

More traditional VPEs (adjectives, adverbs, and nouns) also differ by suggesting internal or external attributions of uncertainties. Ülkümen et al. (2016) proposed that phrases containing “likelihood terms” (*chance, likely*/*likelihood.* and *probability*) primarily describe aleatory probabilities, whose chances depend on objective features of the situation, like frequencies and random variations. In contrast, “confidence terms” (*sure*, *confident*, and *certain*/*uncertain*) are primarily used to describe the knowledge (epistemic state) of sentient beings. Going through two volumes of New York Times they found that likelihood terms were much more often quantified and based on external evidence, compared to confidence terms. The latter reflected more often subjective judgments and were used more often about knowable events of the past. Participants in an experimental study (Ülkümen et al., 2016, Study 3) gave reasons for being X% *confident* by referring to their knowledge, memories, or skills, whereas their reasons for an outcome being X% *likely,* contained more often information about frequencies and other factors beyond the speakers’ control.

In Ülkümen et al.’s (2016) scheme, expectations about singular events were considered epistemic. This set their analysis apart from Kahneman and Tversky’s (1982) taxonomy of “variants of probability”, which allowed the outcome of singular events to be assigned a probability based on factors external to the speaker, like the current strength and weakness of an eligible candidate.

Arguably, some verbal terms, like *certainty*, can be conceived both as internal or external depending on its appearance in a personal or an impersonal clause. While “I am X% certain” describes the epistemic certainty of the speaker, “it is X% certain” presumably reflect external facts (Teigen & Løhre, 2017). A study comparing such expressions showed that people consistently ascribe higher internal than external certainty to the same event; they declared, for instance: “I am 70% certain” that NN would win a race, while estimating that this outcome “it is 60% certain” (Løhre & Teigen, 2016).

### Credibility and expertise

The vagueness and imprecision of verbal terms might indicate a lack of knowledge, compared to numeric estimates, which are accordingly perceived as more trustworthy (e.g., Gurmankin et al., 2004). Numbers suggest that estimates are based on measurements and calculations, reflecting that the speaker is an expert. But this can be turned around as an argument *against* using numbers in domains characterized by high uncertainty. Budescu and Wallsten (1995) have proposed a *congruence principle* for proper use of verbal versus numeric probabilities, which states that the precision of the estimate should reflect the uncertainty of the predicted outcome. According to this principle, a communicator will appear less credible when offering a point prediction for outcomes that are not calculable. But also numeric estimates can be approximate (Ferson et al., 2015), for instance by being described as ranges (e.g., a 60–80% chance), preserving the credibility of the communicator (see also the Credibility and expertise section for outcome ranges below).

Use of high-probability terms and high-probability numbers signals confidence, which people often view as a sign of credibility and expertness, persuaded by “the confidence heuristic” (Price & Stone, 2004). This backfires when feedback on accuracy is available (Sah et al., 2013).

Use of personal vs. impersonal phrases, discussed in the previous section, may affect perceptions of credibility and expertise. Fox and Irwin (1998) suggested that speakers using an internal mode (“I am 70% sure”) appear more responsible for their statements than speakers using external mode expressions (“there is a 70% chance”), and might accordingly be trusted more. But they could also be blamed more if the prediction turns out to be wrong. Results from a recent study indicate that a manager who describes uncertainty in an external way (“it is very uncertain”) is trusted more than one who uses internal attributions (“I am very uncertain”) (Løhre & Teigen, 2022).

### Valence

While numerical probabilities are assumed to be neutral, words may reveal outcome valence. Some terms are positive, in an evaluative sense, others negative, used mainly to describe outcomes that are undesirable. For instance, a *risk* describes, by definition, aversive outcomes. *High risk* can mean that negative consequences are very likely, or very severe, or a combination of both. With specified consequences, for instance death risks or risks of infection, degree of risk simply reflects the probabilities involved. *High*, *moderate*, and *low* risks will in such a scheme indicate levels of probability for the targeted outcome, although it is debatable where they should be placed on a numeric scale. For instance, one health authority (Calman, 1996) has recommended a scale for medical risks, where probabilities exceeding 1% should be called *high risks*, risks between 0.01% and 1% are *moderate*, and only risks of less than 0.01% should be described as *low*. But students and even doctors think “high risks” correspond to probabilities around 40–50% (Berry et al., 2004), whereas “low risks” reflect probabilities in the 5–10% range, in other words risks that according to Calman’s standards are *very high*. Even in a context where exact numeric probabilities are reported, they will affect people’s evaluations and decisions according to their verbal labels. Complications arising from a medical procedure in 2 out of 1000 patients were rated as more aversive when described as *high risks* than as *low risks* (Olchowska-Kotala, 2019).

Desirable outcomes can in contrast be described as *chances*, *opportunities, *and *hopes*. While risks have an external focus, hopes are typically used in an internal and subjective sense; they presuppose *somebody* hoping. They say perhaps more about this person’s subjective states and values than about the actual chances of obtaining a desired goal (Reimann et al., 2014). Yet they can function as VPEs with appropriate intensifiers. A *good hope* will to most people indicate a higher probability of a desirable outcome than a *slight* hope or a *small* one. Hopes are found to have an upper limit, too. It has been shown that prototypical hopes have a medium subjective probability of being attained, ranging from 20 to 90% at various stages of the hope process (Averill et al., 1990). More recent analyses suggest that hopes are more strongly related to people’s *possibility* of attaining a good outcome, than to the strength of expectations or degree of optimism (Bruininks & Malle, 2005; Miceli & Castelfranchi, 2010), particularly in regard to outcomes that are beyond the actor’s control (Bury et al., 2016, 2019).

Most other VPEs, like *certain*, *possible*, *likely,* and *unlikely,* can be used to describe and predict desirable and undesirable outcomes equally well. But some phrases that include negations (see the section on Directionality below) appear improper paired with unattractive events. Statements like: “It is very uncertain you will fail”, or: “It is quite doubtful that you will miss the train”, sound odd. They seem to imply that the individuals addressed *intend* to fail, or, for enigmatic reasons, *want* to miss the train, but may not succeed in their self-destructive endeavours.

### Directionality

Probabilities contain a double message. They indicate that an event may occur, and that something may be the case, but also that it may not happen. Numeric values describe the first aspect of this message more directly than the second. A 70% chance for El Niño to take place this season draws attention to the occurrence of this weather phenomenon, not just because 70% is a high number, but also because the statement is explicitly about El Niño taking place, and not about its absence. We are not told, but can infer, that 70% implies a 30% chance of no El Niño. Verbal phrases can be explicit about either of these events. Some VPEs are focused on occurrences. El Niño is *possible*, *likely*, or perhaps *almost certain*. Other phrases focus on the complementary event: El Niño’s non-occurrence. El Niño may be *uncertain*, *doubtful* or *not completely certain.* Phrases with this flip-side focus may contain a lexical negation (*not certain*) or a negative prefix (*uncertain*). The contrast between these two types of terms have been called a difference in *directionality* (Teigen, 1988; Teigen & Brun, 1995). A VPE of the first kind points “upwards”, towards the appearance of a target outcome, whereas a negative VPE points “downwards”, towards its non-appearance.

Directionality is not to be confounded with *valence*. A “high risk” is *directionally positive* (suggesting chances for occurrence of an aversive outcome (for instance, death). “Unlikely” is *directionally negative*, suggesting non-occurrence of a target event, but might be positive in an evaluative sense, if it implies negation of an aversive outcome (“it is unlikely that you have been infected”).

The concept of directionality was originally introduced to show that VPEs are not simply informal or imperfect substitutes for numbers, but carry other, distinctive meanings of their own (Teigen, 1988). A verbal term’s directionality can be demonstrated by adding an intensifier like “high” or “very”, which implies for positive terms a higher probability (*very likely* is more than simply *likely*) while negative terms are shifted downwards (*very unlikely* is less than just *unlikely*), as illustrated by the standard scales in Table 2.

Directionality can also be revealed by asking people for explanations. It seems that people typically add reasons supporting either an outcome’s presence or its absence, but not both (Teigen & Brun, 1995). Positive VPEs suggest pro reasons, negative phrases evoke reasons against (con reasons). Why has NN *a chance* of winning the tennis match? Because she is a strong and skilful player. Why is it *uncertain* that she will win? Because of her opponent’s skills. Only propositions that explicitly state that it can go both ways (“It is a *fifty-fifty chance*”) include both kinds of reasons (“She is a strong player, but so is her opponent”). The type of reasons people offer can accordingly be used as a directionality criterion. This test reveals that even some low probability expressions (*a risk*, *a chance*, or *a slight possibility*) can be directionally positive, as they evoke more reasons in favour of the target outcome than against it. Directionality can alternatively be determined by asking speakers about consequences. If NN has *a chance* of winning, she is entitled to be hopeful. If it is *uncertain,* she should be prepared for a loss.

Directionality of VPEs has been demonstrated in several languages, including English (Budescu et al., 2003), Norwegian (Teigen & Brun, 1995), French (Juanchich et al., 2010), Chinese (Zhang et al., 2020), and Japanese (Honda & Yamagishi, 2006, 2017). Most phrases that have been analysed this way are directionally unambiguous. Almost all listeners agree that reasons why an outcome is *possible*, *probable*, or *quite certain* should favour a target outcome T, rather than its complement. In contrast, reasons for a *doubtful* or *uncertain* outcome favour its non-occurrence, ~ T. Instances of ambiguity can be found for a few low probability phrases, where positive stem words are combined with qualifiers suggesting smallness. *A small probability* of T is still a probability, and can accordingly be associated with pro reasons for occurrence, whereas its smallness suggests the existence of con reasons. Informal observations suggest that decoupling the qualifier from the stem, “the *probability* of T is *small*,” emphasizes smallness and might accordingly lead to negative interpretations.

Directionality reveals familiarity with language. Zhang et al. (2020) found evidence of a “foreign language effect” for Chinese and English bilingual speakers, who evinced stronger effects of directionality in their native language than in their second language.

The directionality of numerical expressions can be investigated in the same way. Sentences like: “The team has a X% chance of success, because …” were normally, but not exclusively, completed with pro reasons (Teigen & Brun, 2000), so with respect to directionality, numerical probabilities appear to be more ambiguous than words. High *p* values led generally to more pro reasons than low values, but even for a 30% chance, participants gave pro reasons as often as not. In the case of undesirable outcomes, pro reasons were in majority for still lower probabilities.

Directionality can be regarded as a kind of *framing *of probabilistic statements, comparable to describing a glass of water as “half empty” or “half full” (McKenzie & Nelson, 2003). One might assume that positive phrases describe primarily events that are expected to occur, whereas negative phrases are more apt to describe low probability events, or, more generally, that directionality can be predicted from their membership functions on the 0–1 probability scale (Budescu et al., 2003). In line with this, most standard scales use negative terms only to describe low probability events, as illustrated in Table 2. Despite this, it is possible to create lists where positive and negative VPEs are more evenly distributed over the (0,1) span (Honda & Yamagishi, 2017; Juanchich et al., 2010; Piercey, 2009; Teigen & Brun, 2003b), and thus obtain alternative frames both for high, medium and low probability outcomes.

If directionality is not simply determined by probability, what makes a speaker frame a specific outcome in positive or in negative terms? Research on framing indicates that frames are determined by reference points and by the speaker’s communicative intentions. McKenzie and Nelson (2003) showed that glasses that were about to be filled up were described according to how *full* they were, but those that had been initially fuller, were described as partially *empty*. The way speakers frame their messages will accordingly “leak” information about trends relative to a previous state of affairs (Sher & McKenzie, 2006). Similarly, a probability that has been revised upwards, for instance from 30 to 60%, will typically be described in positive terms as “probable”, but one that is revised from 90 to 60% will be described negatively as “not certain” (Juanchich et al., 2010). Negative VPEs were chosen when an interlocutor had expressed exaggerated chances, while positive expressions were preferred in response to estimates that were too low. More generally, the type of VPE chosen depends upon the speakers’ situationally determined reference values (Honda & Yamagishi, 2017).

Directionality does more than priming positive or negative reasons for an outcome. They also convey attitudes, recommendations, and beliefs, and may facilitate different decisions. Teigen and Brun (1999) described a patient, Marianne, who was considering a new and controversial treatment for migraine. Participants in one condition were informed that the treatment had “some possibility” of being helpful. Nearly all said they would advise Marianne to try this cure. In a second condition, they were informed that it was “quite uncertain” it would be helpful. In this group, only 1/3 of the participants would recommend the cure. But both these phrases were assumed to describe probabilities in the 30–35% range, so the difference in recommendations could be attributed to directionality of verbal phrase. Participants in a third condition, who only received numerical probabilities, were more split: 58% recommended treatment, and 42% advised against it. Thus, when it comes to recommendations and warnings, numbers can be more ambiguous than words.

Similar results have been found for verbal vs. numerical forecasts of investments. A message containing the term *unlikely* was rated higher in clarity than a corresponding numeric chance (25%). The authors conclude that “verbal probabilities convey implicit recommendations more clearly than probability information, whereas numeric probabilities do the opposite” (Collins & Mandel, 2019, p. 683). Comparing numeric and verbal forecasts of a flood, Jenkins and Harris (2020) showed that directionality predicted ratings of correctness and surprise more strongly than high and low *p* values did.

Directionality impacts the way messages are combined. Mislavsky and Gaertig (2021) found that verbal statements reinforce each other, so if two financial analysts both say that stocks are “rather likely” to be profitable, recipients infer that an increase in value is “quite likely”. It turns out that this increase in certainty requires positive phrases. Negative phrases (both advisors say a rise is “not completely certain”) make people think that the combined chance is lower (Teigen et al., 2022b).

The usages and effects of positive versus negative VPEs are not symmetrical. Phrases with a positive directionality are more numerous and offer a larger lexicon of different terms than corresponding negative phrases. Lists of VPEs in “translation studies” typically contain twice to three times more positive than negative terms, and studies of people’s spontaneous characterizations of chance events display an even larger predominance of positive terms (Budescu et al., 1988; Teigen & Brun, 1995). Positive phrases can be used in a more neutral sense than phrases with negations. They serve more often as labels for the full scale or dimension; we ask people to rate how *likely*, how *certain*, or how *possible* an outcome is, rather than how *unlikely*, *uncertain*, or *impossible*. Thus, positive terms belong more often to a broad class of *unmarked* (neutral, default) terms, as defined in linguistics (Battistella, 1996; Clark & Clark, 1977). Their negative counterparts are typically “marked” with prefixes or negations, which reveal their secondary status. Linguists have suggested that negations convey a mixed message, presupposing a tacit proposition that is then denied (Horn, 1989).

## Uncertainties as outcome ranges

A highway project is predicted to take from two to five years to complete, and to cost between 90 and 180 million dollars. Projections of global warming in this century range from 1.5–5.0 ℃, and medical authorities believe that 5% to 15% of heavy smokers will develop lung cancer. What is communicated by such approximators? How certain are these intervals? Such range estimates are well known by risk analysts as probability intervals, credible intervals, or confidence intervals, with distinct technical meanings. In the present context they will not be formally defined and distinguished from each other but will be called uncertainty intervals as an umbrella term for all kind of ranges.

### Degrees and levels of uncertainty

An obvious feature of ranges is their width. With increasing knowledge, the estimated endpoints become closer to each other; the project is predicted to take 3–4 years instead of 2–5, and to cost 120–150 million instead of 90–180 million. The width of an interval can accordingly be regarded as a measure of uncertainty. Receivers feel that narrow ranges are more informative and convey more knowledge and certainty than wide ones. Communicators are faced with a trade-off between precision and accuracy (Yaniv & Foster, 1995, 1997). Should they appear informative at the expense of accuracy, or suggest a wide interval to maximise their hit rates while being accused of vagueness?

Probabilistic estimates appear to offer a solution. A forecast can be conceived as a probability distribution with confidence intervals spanning 90% or more of the potential range of outcomes. To make the interval 100% complete requires a much bigger span than one that is merely designed to contain expected, “normal” outcomes. This gives us two contrasting, but complementary measures of uncertainty: width of range (wide vs. narrow) and associated degree of confidence (low vs. high). Formally, these measures are inversely related, so a narrow interval that contains, for instance, 60% of the distribution could be compatible with a 90% interval that is perhaps twice as large.

Lay people seem often to mix up these two indicators of uncertainty. Some people realize (correctly) that narrow intervals entail less confidence, whereas less numerate participants believe that narrow intervals and high probabilities go together (Løhre & Teigen, 2017). Wide interval projections about a rise in global temperatures were generally seen to “convey more uncertainty,” and were considered less informative than narrow intervals. At the same time, they were judged by a majority as “more certain to be correct” when they were wide (Løhre et al., 2019a). Questionnaire results indicated that it was easier and more intuitive to associate intervals with uncertainty than with correctness.

### Hedging

Verbal qualifiers like “about” and “around” are often added to numeric estimates to indicate that they are approximations and should not be taken literally. Numbers without decimals and round numbers signal a similar “aboutness” and convey estimates that should be taken with “a grain of salt” (Ferson et al., 2015). Ranges are typically bounded by round numbers; weather forecasters predict 10–30 mm, not 9–31 mm of rain. Such estimates are easily processed and appear less definite, especially when qualified by verbal phrases like “expected” or “most likely”.

### Sources of uncertainty

An outcome interval can reflect external as well as internal processes. Wide intervals may indicate actual variability in the external world, or originate in one’s ignorance surrounding one specific factual item. Interestingly, these sources may lead to different evaluations of estimates. Wide interval estimates of a variable quantity (for instance, the price range of a flight ticket) may reflect accurate knowledge of the domain in question, whereas wide interval estimates for a stable quantity (for instance, the distance to the destination) indicates lack of knowledge. Such judgments will, in turn, affect judgments of expertise and interpretations of lower and upper interval bounds, as discussed in the next two sections.

### Credibility and expertise

An early study of the “preciseness paradox” (Teigen, 1990) found that a precise estimate was deemed to be more believable than statements about a wider interval that actually contained the narrow one. However, when participants in a parallel condition were asked which statement they would be more *sceptical* about, they chose again the narrow estimate, which now appeared overly precise.

Thus, the credibility of preciseness depends on the question asked, together with the assumed predictability of different domains (Du et al., 2011). Most people expect precision to be attainable in some areas (e.g., in medicine), but not in others (e.g., political forecasting). In domains with high variability, precise predictions may be viewed as less trustworthy than vague ones. Joslyn and LeClerc (2012, 2016) showed this to be the case for weather forecasts and climate projections, where “deterministic” (point) predictions were trusted less than estimated prediction intervals.

When people are asked to indicate their own prediction intervals, they rarely ask how “confident” they are supposed to be. Software developers who gave work time estimates for various tasks produced equally wide intervals in 90%, 75% and 50% confidence conditions (Teigen & Jørgensen, 2005). Similar results were found for general knowledge questions where people typically became “overconfident” (or rather too precise) when instructed to give 90% intervals, but not when they were supposed to generate 60% or 30% intervals (Langnickel & Zeisberger, 2016). A recent set of studies showed that they did not distinguish between a “likely” and a “most likely” interval in terms of width. They also estimated both intervals as equally probable (Teigen et al., 2022a).

When people receive interval estimates of a continuous variable, they often fail to understand the meaning of an arbitrary placement of upper and lower bounds delimiting a section of the underlying bell-shaped probability distribution. They often seem to think that all outcomes within an uncertainty interval are equally likely (Dieckmann et al., 2015). When actual outcomes are disclosed, all outcomes that fall within this range are typically judged as correctly predicted, with a sharp drop for outcomes above the upper or below the lower bound. Indeed, outcomes situated exactly at the upper or the lower bounds were deemed to be accurately predicted, regardless of the confidence associated with the interval (Teigen et al., 2018b).

### Single-bound estimates as comparative judgments

Uncertainty intervals can be incomplete in yet another way. A climate scientist may predict a temperature increase of “at least 1.5 degrees” instead of offering the full range from 1.5 to 5.0 degrees. The meanings of such “one-sided” or partially bounded uncertainty estimates have rarely been explored, despite their common occurrence. For instance, we do not know whether people think such estimates are more, or less, informative and trustworthy than complete intervals, although it appears that worst-case scenarios in climate predictions are trusted less than complete ranges (Howe et al., 2019). Participants in this study also received most likely (point) estimates. Single upper or lower bounds are otherwise, when taken literally, not informative of most likely values or interval width. “At least 1.5 degrees” could, in principle, mean any temperature from 1.5℃ and upward towards infinity, but pragmatic rules of conversation, in this case Grice’s (1975) maxim of quantity, indicate that values rather close to 1.5℃ (e.g., 1.7℃) are expected. A speaker with a much larger temperature increase in mind would suggest a higher single bound, to optimize amount of information.

Upper and lower bounds are described by a combination of numerical and verbal terms. A distinction can be made between those delimiting inclusive and exclusive intervals. *At least*, or *minimum* X degrees include X in the prediction interval, whereas *more* than X, or *over* X, imply that X is outside of the interval and will not occur. For upper boundary estimates, *at most* and *maximum* are inclusive and *less than* or *under* are exclusive. Intervals delimited by inclusive boundaries will normally be smaller and perhaps appear more exact and trustworthy (Teigen et al., 2007a). But this applies primarily to epistemic (internal) uncertainty, as for instance a fixed, but imprecisely known distance between two cities. When uncertainty is due to external variability, as for instance ticket prices for a flight between the same two cities, inclusive min- and max-estimates yielded, perhaps surprisingly, *larger* intervals than exclusive more than/less than-estimates (Teigen et al., 2007a, Exp. 3 and 4). We do not know whether they would also be judged more trustworthy or indicate a higher degree of expertise.

### Directionality again

Single limit statements carry an implicit communicative message in addition to their literal numeric meaning, much like the directionality of verbal phrases. *At least*, *minimum* and *more than* point upwards and indicate that we are talking about something large. If prices for a product lie in the $100-$150 range, a speaker might say that it costs *at least* (or *more than*) $100, or, alternatively, *less than* $150. These statements are not neutral. The first suggests that the product is expensive, the second that it is affordable. So even if the lower limit estimate is the smaller of the two, it functions as a reference point and implies largeness, whereas the upper limit calls attention to the target object’s relative smallness. Thus, choice of lower versus upper limit estimates can reveal the speaker’s attitudes, recommendations and concerns (Teigen et al., 2007b). A lecture attended by “more than 100” students suggests a crowd that almost filled the hall, whereas an audience of “less than 150” suggests a number of empty seats (and, by implication, a larger lecture hall).

Speakers using boundaries framed in the same way appear more in agreement with each other than speakers choosing opposite boundaries, even for estimates that are compatible. Similarly, experts adjusting their former estimates up or down were considered more consistent when both estimates were of the “more than”- type than when the second estimate was framed in terms of “less than”, suggesting a change of opinion (Løhre et al., 2019b).

Single limit statements can also suggest trends. When people are told that tomorrow’s temperature will be “above X degrees” they think it will be warmer than today. If it will be “below Y degrees” tomorrow, they think it will be colder (Teigen, 2008).

Lower and upper limit statements are not used equally often. Frequency counts show that *more than-*statements vastly outnumber *less than*-statements for comparable amounts of money, distances and durations (Halberg & Teigen, 2009), except in the case of very small numbers. The effect is not limited to numeric estimates. *More than*-statements are preferred to *less than-*statements even when describing logically equivalent relationships. They lead to more agreement and will more likely be considered true (Hoorens & Bruckmüller, 2015; Zhang & Schwarz, 2020). This asymmetry may reflect an even more general preference for comparing objects in terms of which is *larger*, *higher*, *stronger*, *wider,* and *fuller* instead of *smaller, lower, weaker*, *narrower,* and *emptier* (Matthews & Dylman, 2014; Skylark et al., 2018). This “Higher Use of Larger Comparison (HULC)”-effect may in turn be related to the concept of linguistic markedness, which implies a tendency to name dimensions after the upper, “unmarked” end of the dimension (Battistella, 1996; Clark & Clark, 1977).

Relatedly, by being the default option, *more than*-statements seem more neutral than corresponding *less than*-statements. An academic who has published “more than 30 papers” appears productive, but “more than 30” might just be a rough estimate when exact numbers are not needed or not known. “Less than 40 papers” would, in contrast, clearly be derogatory and makes us think that a higher number was expected. Such upper limit statements function linguistically as a shortfall, supposing a larger reference amount that is not attained (Moxey et al., 2001; Sanford et al., 2007).

**Fig. 1:**
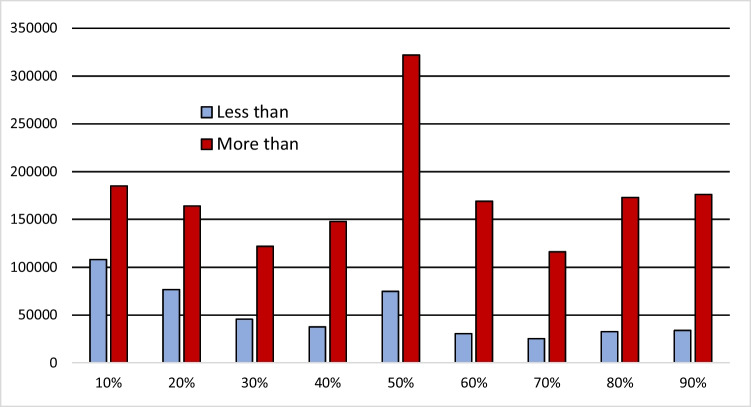
Occurrence frequencies of “less than X percent” and “more than X percent” for X = 10–90% in Google News (February 2020)

Percentages and probabilities are commonly described as *more* or *less* than a prominent round number. We say “more than 60 percent” but rarely “more than 59 percent”. More (or less) than 50 percent is particularly frequent. Figure 1 shows frequencies in Google News for *more* or *less* than X percent (not limited to probabilities). While *more than*-statements are numerous throughout the range, *less then*-statements are more specific by being limited to low percentage values. Similar counts of probability expressions show that “More than X percent chance” is applicable to both likely and unlikely outcomes, whereas “Less than X percent chance” is reserved for chances of 50% or below (Hohle & Teigen, 2018, Fig. 1).

The directionality inherent in more than- vs. less than-estimates is parallel to directionality of verbal phrases discussed in a previous section and can be tested with similar methods. Hohle and Teigen (2018) asked people to suggest reasons for why a glacier had “over 30% chance” of attaining half its present size in the future, and received mostly pro reasons for melting, whereas those who were told that the chance was “under 50%” gave mostly reasons for melting slowing down (con reasons). Participants were also asked to rate appropriateness of selected verbal phrases to describe outcomes that were either “more than” or “less than” 30, 50, or 70% likely. *Possible* was appropriate in more than-statements, while *uncertain* was more appropriate in less than-statements.

### Quantities suggested by verbal phrases

When VPEs are used to describe the outcomes of continuous variables, their meanings seem to change. Instead of characterizing outcome probabilities, they highlight a location on the outcome scale. So, if a test of 100 batteries shows that they last from 1.5 to 3.5 h with normal use, people will select 2.5 h as a *likely* (*probable*) duration for a battery even if the occurrence frequency of this specific outcome may be much less than 50%; in other words, they answer as if “probable” and “average” or “most likely” mean the same. This way of asking people to describe VPE usage rather than probabilistic meaning, was introduced in an investigation of *will* and *can* (Teigen & Filkuková, 2013). A battery *will* last how long? 1.5 h. A battery *can* last how long? 3.5 h. These answers indicate that “will” evokes an *at least- *interpretation, and “can” is used to signify *up to* or *at most.*

This novel way of study meanings in “Which outcome” studies has revealed a *centrality effect* for “likely” (Teigen et al., 2022a) and an *extremity effect* for many other verbal phrases. Respondents in these studies selected outcome values that did not match the probabilities in standard guidelines, like those displayed in Table 2, or numerical equivalents generated by a conventional “translation” design. Thus the lowest battery duration was picked to illustrate an outcome that was *certain* (like *will*), and the top value, 3.5 h, was selected to illustrate a *possible* outcome (like *can*), even if as few as 5–10% of all the batteries lasted that long (Juanchich et al., 2013; Teigen et al., 2014). People also selected high values to indicate what has *a chance* to happen, is *uncertain*, is *possible*, and *may* or *could* be the case (Teigen et al., 2018a, 2018b). An *entirely possible* outcome is in this context not an outcome with a probability around 50%, as some translation studies indicate (e.g., Lichtenstein & Newman, 1967; Reagan et al., 1989), but rather the topmost outcome, which in a bell-shaped distribution has a very low probability of occurring (Teigen et al., 2019). When asked to describe an *unlikely* or *improbable* outcome, speakers typically selected an extreme value that had yet to be observed, located outside of the range of expected results. If the ocean level is supposed to rise 50–90 cm, an *improbable* rise is 1 m (Teigen et al., 2013). The numeric probability of such an outcome will be close to zero, rather than the 15–30% probability that typically is suggested as corresponding to *unlikely* and *improbable* in standards for recommended use (cf. Table 2).

Both the *centrality* effect (of likely) and the *extremity* effect (for other VPEs) are quite robust and appear to persist even when participants are given graphs displaying probability distributions or receive numerical explanations of what the terms are supposed to mean. Jenkins et al. (2018) recommend accordingly that numbers should be given first, with VPE appended (the N-V method), instead of adding numbers to “explain” the verbal phrase (V–N).

## General discussion

This paper goes beyond most other discussions of uncertainty communication, by reviewing findings of two kinds: from studies describing probabilities of single (binary) outcomes, and from research on complete or incomplete outcome ranges of continuous (multiple outcomes) distributions. In both domains, uncertainties can be expressed by means of words and numbers, or by a combination of both. Strictly speaking, *a 30% probability* combines a number, *30*, and *probability*, which is a verbal term. Similarly, the end points of an interval, described by numbers, are turned into combined phrases by including verbal qualifiers like *more than* or *at least*. All these types of statements give information about what speakers think is the case and what hearers might expect, along with indications of the limitations and imprecision of this knowledge. They can accordingly be viewed from two perspectives, as statements of knowledge or as admissions of ignorance, and be discussed under alternative headings as expressing either *probabilities* or *uncertainties,* depending on perspective.

The two settings compared in this review are similar not just in addressing probabilities and uncertainties in factual, explicit and descriptive ways, but also, and more indirectly, by including hints about the speakers’ attitudes, their communicative intentions, and their persuasiveness and credibility. They also suggest the origin of the uncertainty, the valence of the target outcome, implicit reference points, and the direction of potential trends. We have in this article especially emphasized and explored *directionality*, that is, complementary ways of directing listeners’ or readers’ attention towards occurrences or non-occurrences of single outcomes, or towards the largeness or smallness of quantities measured on a continuous scale. We find in both settings evidence for positive/negative asymmetries but also disclose some paradoxical shifts in meanings for common verbal probability expressions. The challenge of uncertainty for practitioners is to be aware of such non-probabilistic features and not believe that they are permanently solved by prescriptive definitions of what verbal or numerical expressions should technically mean.

### Implications for communication

Risk analysts and other experts are often required to be “neutral” in their communication to decision-makers and in statements intended for the general public. The research reviewed in the present paper shows that ideals of objectivity and neutrality may be hard to achieve. A probabilistic message must be framed in one way or another and will accordingly convey a corresponding emphasis on probability or uncertainty. The way it is phrased will further suggest implicit comparisons, and reveal evaluative attitudes, like reassurances, recommendations, and reasons for concern. Narrow uncertainty intervals may be chosen to maximize informativity, but at the risk of being inaccurate.

We think it is important for communicators to be aware of the pragmatic connotations that surround all estimates, as surveyed in the present paper. For instance, defining “likely” as corresponding to probabilities above 2/3 might look like a rough and handy approximation, whereas p > 66% (as in the probability scales displayed in Table 2) may appear too specific to be credible, although the two numeric expressions are interchangeable from a mathematical point of view.

Some frames, or modes of expression, are more neutral and open for different interpretations than their counterparts. A climate scientist who predicts an increase in sea level of “more than 50 cm”, may want to tell that we can expect a large, perhaps alarming, rise. But the lower limit of 50 cm could also have been picked as the nearest round number, or because the upper limit of the increase is not known. A single upper bound, as for instance “less than 100 cm” would appear odd.

For proper understanding, it is essential to know the conversational setting under which the estimates have been produced. Is the frame and format freely chosen, or does the estimate come in response to an issue raised by someone else than the speaker? In a discussion about implausible estimates (Priestley et al., 2021) a climate scientist might use a “less than”-statement without implying that the rise is insignificant. However, when cited out of context, the “less than”-statement might appear callous. Similarly, the phrase “not certain”, could simply come as an appropriate qualifier of somebody else’s point prediction. But quoted out of context, it might be taken to suggest that the experts are just guessing. To avoid such interpretations, communicators should embed their estimates in full sentences, providing their own contexts, and make a habit of phrasing their message in more than one way, for instance, by saying “it is likely, but far from certain”, or that the sea level will “very likely (*p* = 0.90) rise with 60–90 cm, but a lower or a higher rise cannot be ruled out”.

Similarly, phrases from a standard scale, like those displayed in Table 2, make only sense for recipients who are aware of the alternative phrases that are *not* chosen. An intelligence report stating that a terrorist attack is “unlikely” will reassure most readers, except those who notice that the intensifier “highly” is missing (this modifier should have been included if the risk was close to zero). In general, one should make clear when phrases are used in a technical, predefined sense, and when it is just used as part of everyday language. To avoid that these usages are not mixed up one might as well replace the verbal terms that form a part of ordinary conversations with a schematic display of risks of different levels (or colours).

### Concluding remarks

Presenting verbal probabilities and outcome ranges side by side, as attempted in this paper, reveals some common themes but also differences and lacunae in our knowledge. It seems that many authors have struggled with coming up with umbrella term for what these modes convey. Many overviews of verbal and numerical expressions are titled “communication of uncertainty” (e.g., Dhami & Mandel, 2022; Juanchich et al., 2019; Kahneman & Tversky, 1982; Ülkümen et al., 2016; Van der Bles et al., 2019), despite primarily discussing probabilities, not uncertainties, and rarely offer an analysis of how these two concepts differ from each other. First, they differ in directionality, *probability* being a positive and *uncertainty* a negative term. Second, probabilities typically refer to specific outcomes (or a class of outcomes), whereas uncertainties suggest a plurality of alternative options, or multiple outcomes, like the intervals discussed in the range section of this present manuscript. While probabilities are assumed to reflect the strength or frequencies of outcomes, uncertainties indicate which or how many outcomes to expect. Thirdly, they are not co-extensive in the sense that they span a full scale defined by polar opposites. The upper endpoint of a probability scale is often defined as 100% certain, whereas the lower endpoint, zero probability, is not equivalent to full uncertainty. Instead it is explained as *impossible* – suggesting yet another concept. Perhaps *expectancies* would be a better and more comprehensive term for capturing all three dimensions, with *uncertain* indicating p < 1 and *possible* p > 0. To date, few studies have been conducted to compare estimates of probability and certainty. One study showed a gambling wheel with sectors occupying different proportions (Teigen, 1994). When asked to estimate the *probability* of their chosen sector occur, almost all (97%) participants answered by reporting (correctly) the proportion. When participants in a different condition were asked to estimate how *certain* they were, the frequency of normative answers was much lower (57%). Further studies should compare probability estimates and range estimates in a more systematic way, for instance whether Kahneman and Tversky’s (1982) four-fold scheme of probabilities (external/internal; frequency/propensity) is equally applicable to range estimates. A recurring paradox is the finding that people use verbal phrases to describe infrequent outcomes in a range or a set of outcomes as “unlikely”, “possible”, and even “likely”, and yet translate them into numeric probabilities that are much higher than these usages should indicate. Such inconsistencies suggest that people alternate between several concepts of uncertainty, even in the same session, according to the elicitation method used. This makes them flexible thinkers but perhaps less reliable advisors.
